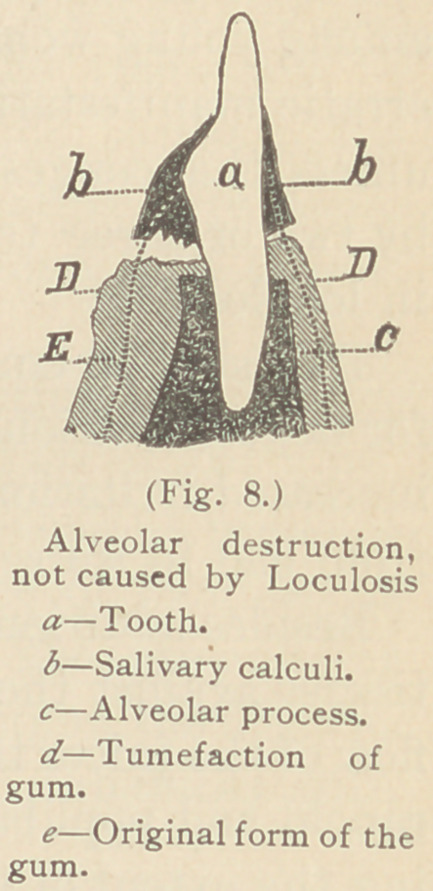# Loculosis Alveolaris

**Published:** 1886-09

**Authors:** J. N. Farrar

**Affiliations:** New York City


### Independent Practitioner.

VOL. VII. SEPTEMBER, 1886. NO. 9.

### Original Communications.

NOTE.—No paper published or to be published in another journal will be accepted for this department. All papers must be in the hands of the Editor before the first day of the month preceding that in which they are expected to appear. Extra copies will be furnished to each contributor of an accepted original article, and reprints, in pamphlet form, may be had at the cost of the paper, press-work and binding, if ordered when the manuscript is forwarded. The Editor and Publishers are not responsible for the opinions expressed by contributors. The journal is issued promptly, on the first day of each month.

### LOCULOSIS ALVEOLARIS

(POCKET DISEASE OF THE ALVEOLUS.)

FOUR STAGES.

(Continued from page 343.)

BY J. N. FARRAR, M. D., D. D. S., NEW YORK CITY.

A LECTURE DELIVERED BEFORE THE BROOKLYN DENTAL SOCIETY, FEB. 8, 1886, ACCOMPANIED WITH BLACKBOARD DRAWINGS.

### III.

*General Remarks.*—The treatment of the subject of Loculosis, from the initiatory stages to the final result of an unchecked career, covers a wide range.

So far as I have investigated, there appears to be some half a dozen or more causes which may contribute to the establishment of the disease. It is not my present purpose, however, to enter minutely into details, but at some future time I may express my views more fully through some of the periodicals.

Although I shall not deal with the tempting phases of the deeper causes, I would not have it thought that my remarks are empirical or one-sided, for they are based upon careful practical investigation.

To begin I will forestall the conclusion somewhat, by stating that the rise and progress of this disease, which *once established is never cured by unaided nature except by ejection of the tooth,* may be divided into four stages: 1st, gingivitis; 2d, separation of the lining membrane of the socket from the root, constituting the pocket; 3d, caries; 4th, necrosis of the alveolus.

I will outline the progress of an extreme case, noting its career from incipiency to the worst stage, as it appears to the unaided eye and sense of touch. In doing this I shall disregard some of the speculative notions in vogue, which are based on assertions that have no foundation in fact.

While I am convinced that this disease is the combined result of systemic tendencies and local excitants, I shall confine my remarks to the latter; not following, however, the notion which has more than once been advanced, that "it arises from the same cause that leads to exostosis," an idea based on the ground that because excitement of the tissues causes the latter, and is common to both in their earlier stages, it consequently must be the cause of the former, for that would be about as logical as to assume that because "festering" around a sliver accidentally driven into the foot and bunions are both preceded by local excitement of the tissues, the primary cause of both must be of the same kind.

Without going into histological details, let it suffice to say that the direct cause of exostosis is generally irritation of the coverings of the root, through some violent action of the tooth, as from the habit of biting threads, while on the other hand, the causes of loculosis, local, as well as systemic, lie outside of the tooth, starting from the peculiar condition of the system which, in a nut-shell, may be said to impregnate the circulatory and oral juices with earthy matter to such a degree that the immediate environments of the tooth, such as decomposed food and altered secretions resulting from perverted functional conditions of the tissues immediately in contact, cause a chemical precipitate upon the tooth of the calcareous matter that floats in these circulatory and oral juices, to which state of things the naturally susceptible constitution of the alveolar ridge, which is one of the most easily effected parts of the body, is a powerful assistant. Let us trace the local.

(Fig. 7.)

Sectional view of a case of Loculosis, showing two pockets and the calcareous accretions upon the roots of the tooth.

As everyone is aware, there is no union of the gingival margins of the gum with the enamel; therefore, as the gum in its normal condition generally extends above the point of its attachment, slightly overlapping the enamel, forming a shallow trough around the neck of the tooth, although closed by contact while healthy it will be seen great harm is liable to ensue should irritating matter get into it.

While the symptoms of gingivitis and the earlier symptoms of loculosis are the same, and gingivitis seems always to precede loculosis, still gingivitis is not always a forerunner of loculosis; in other words, a symptom does not always imply disease; a cough does not always indicate phthisis pulmonalis.

*Gingivitis, 1st stage.*—The first characteristic of the true forerunner of loculosis is a peculiar redness of the margin of the gum, which once seen cannot be forgotten. The lip has not a surface rawness of the tissues, like that caused by collections of "salivary calculus" upon the *crowns* of the teeth (Fig. 8), but a disturbance within—the lip still retaining its epithelium, which condition is indicated by a deeper red, bordering upon the blue, and on becoming chronic gradually changes to a lighter and somewhat translucent shade.

(Fig. 8.)

Alveolar destruction, not caused by Loculosis

*a*—Tooth.

*b*—Salivary calculi.

*c*—Alveolar process.

*d*—Tumefaction of gum.

*e*—Original form of the gum.

Although retaining the epithelial covering about the neck of the tooth, this ring becomes more and more pronounced in outline as the disease advances, until it passes the line of mere inflammation and becomes incipient loculitis, with a pyogenic surface on the inside.

Careful examination within the annular trough detects a small quantity of decomposed food mixed with rancid serumal juices, and generally, if not always, in addition a small quantity of rough calcareous matter fixed upon the surface of the root, the nature of which is similar to that found upon the crowns of teeth, but generally more dense and of a darker color. At this earlier stage it is a mixture of two varieties of deposits, both from the blood; one directly, having oozed through the soft tissues constituting the walls of the trough, the other by a roundabout way through the salivary and oral glands. The first is called serumal deposit and the second salivary.

As the disease extends down the root, the per cent. of the serumal deposit increases. If the soft deposits are irritating, the rough calcareous accretions are more so; one acting not entirely unlike a rancid poultice, the other traumatically like coarse dried sand paint, which pricks and chafes the soft tissues of the trough every time the tooth moves, if loose, or when the tongue or food presses upon the outer surface of the gum. This aggravates and increases the congestion, causing the lip in effect to pouch out from the lingual and buccal surfaces, the disease extending down between, so that the tissues bleed from slight cause.

Probably the most pronounced cases of congestion are found among young women at a period when the whole system is liable to erratic manifestations; but, fortunately, because of their youth, and unless the congestion is too far advanced, they are easily cured by two or three treatments, and it is not liable to recur, or to end in loculosis.

This swelled condition of the annular lip increases the capacity for deposit, which accumulates as fast as room is made. In this way increased irritation and enlargement of the trough go hand in hand from bad to worse.

Besides additional deposits from without, the chafing of the soft tissues against the roughness within causes an increased osmotic flow of the juices through the walls into the trough, which, becoming rancid and populated with microbes, make matters worse by increasing the extent of the pyogenic surface, leading to formation of small quantities of pus.

Although now constituting the walls of a filthy nest of microbes, the diseased tissues at this stage are easily cured by thorough cleansing, followed by injection of some germicide lotion; or even if left in blood, if carefully watched, to keep away the exciting cause, with an eye also to the original tendencies, possibly the disease may never recur; but once loculosis has gained a strong foothold, even if cured, it is liable to recur from slight cause, for it should be remembered that while the removal of the exciting cause may cure the local disease, it does not do away with the causes which underlie the local excitants.

*2d stage.*—If this irritating deposit is allowed to remain, the quantity will increase as the increasing irritation enlarges the receptacle, and *vice versa.* In this way the territory of the mischief widens until the congestion and stagnation cause the lining membrane along the line of its union with the root to become so weak that it finally falls away. Thus begins the formation of the true pocket, which, from the same cause, goes on increasing in depth and size until a pronounced type of the disease is established. Fig. 7 illustrates the appearance of two sizes of pockets often found at this stage.

If the disease continues unchecked, the hard tissues underneath become affected, and waste by retrogressive metamorphosis, urged on by deeper inflammation, so that the gum margins recede. In one sense this retrogression is a sort of physiological act on the part of nature, for the purpose of ridding itself of the irritations by undermining them, or in other words to reduce the socket below the deposits, even at the expense of the tooth. Nature here seems to work on the same line of argument as did the man who burned his barn to get rid of rats.

At this stage, or a little before, there is sometimes an absence of epithelium around the tooth exposing a raw surface of the gum, wasting of the gingival margin of the gum, or even the presence of this raw circular tract, although strong evidence of loculosis is not always a proof of it, for both may result from the presence of large quantities of rough calcareous accretions upon the crowns projecting over the gums, where all manner of inhabited filth may collect. (Fig. 8.)

Besides the degenerated vitality of the soft tissues at the bottom of the pocket, there is probably some influence contributing to increase the depth of the pocket, through the impaired vitality of the cementum along the line of its union with the socket membrane.

To reiterate, but without entering, as before said, the domain of constitutional influences underlying this disease, the principal secondary cause of the separation of the socket membrane from the tooth, in a nutshell, seems to be congestion of the blood tracts caused by foreign substances wedging along, so to speak, between the socket membrane and the root of the tooth, as fast as the separation takes place.

Although generally confined to narrow limits and to one or two places on one side of the tooth, the disease occasionally attacks several points on one or more sides, often more or less circumscribing the root in one direction or the other, or both; sometimes cutting off the nutrient supplies to the dental pulp, and occasionally of the septums between the sockets to such an extent as to involve the adjacent socket membrane.

At this stage, or a little earlier, some patients experience disagreeable sensations about the teeth, even to pain; but pain, *per se*, although an evidence, is not always proof of any special stage. While most people begin to experience uneasiness at this stage, others are so sensitive that they feel something wrong much earlier. On the other hand, there are those who never seem to experience any inconvenience, even if their teeth are loaded with calculi and stand in inflamed pockets of pus.

While all this mischief is going on, some of the calcific elements of the alveolar process behind the soft tissues continue their retrogressive changes and are absorbed. This increases the diameter and reduces the depth of the socket. Sometimes entire septums between the sockets are in this way destroyed. If the alveolar process could be macerated and dried while in this condition, it would appear soft and porous like that portion of the alveolar process around a large abscess. This perverted condition may be carious, but not necessarily necrotic.

We have now arrived at a point beyond which the disease seldom advances, except perhaps in the degree of waste of the tissues, which generally goes on undermining the tooth until it falls out; after which the socket wall becomes covered with new granulations, perfecting into tissue, and the local disease rapidly disappears.

At this stage cure is somewhat more difficult than at the previous stage, and the disease, if cured, is more liable to recur, for the reason that the surface of the root which is exposed in the pocket is often so low in vitality that union is too feeble to resist future collections upon the teeth, even though slight in quantity, but more because death of the surface portions of the cementum prevents any union whatever. While in a majority of cases the disease, *per se*, can be cured by careful cleansing of the parts and injecting some such germicide drug as diluted non-escharotic creosote, at the same time letting the medicine overflow into the mouth sufficiently to destroy all of the oral microbes, the exposed portions of some of the roots are so degenerated and softened, even upon living teeth, that cure is impossible unless it is scraped away. I speak emphatically upon this point, because experience has taught me its importance.

It may be thought by some people that unless the pocket is closed by reunion of the soft tissues with the root, the disease cannot be said to be cured; this would be as erroneous as to think that unless an eye that has sloughed out by some kind of local disease, which subsequently disappeared, is not restored to the socket, the disease cannot be said to be cured. The pocket is the result of the disease, but the pocket is not the disease itself. To be sure, the union of the soft and hard tissues is desirable, and is frequently attained in practice, but to assume that loculosis cannot be said to be cured so long as the soft tissue has not united with the root, would be as wrong as to assume that it cannot be cured if by extraction there should be no root left for it to unite with.

*3d* (*Carious*) *stage.*—In proportion as the disease becomes violent, it interferes with the nutrient supplies, not only to the tissue involved, but to the adjacent tissues, whether hard or soft, causing a fall in tone of vitality; sometimes this influence is so great that the hard tissues take on a character analogous to mortification of the soft tissues. In short, the alveolus is struggling with caries. At this time, and occasionally during the stage immediately preceding, particles of calcific matter become detached and work themselves through the gums, causing, in appearance, a sort of gum-boil (not alveolar abscess). But although lower in vitality than the second stage the case now, unless too far advanced in disease, is generally curable without excision of the hard tissues.

The treatment is plenty of nutritious food, thorough cleansing of the pockets, followed by some stimulating non-escharotic, antiseptic germicide. If the hard tissues have degenerated beyond resolution, excision becomes necessary; but experienced judgment only can draw the dividing line.

*4th (Necrotic) stage.*—Increased quantity of pus from the pyogenic surface added to the carious condition of the hard tissues behind the pericemental wall of the pocket, accompanied by still greater congestion, impairs the vitality of the parts to such a degree that the stagnant condition of the nutrient apparatus is so great that some portions of the sick tissues die from starvation and poison.

At this period separation between the soft and hard tissues in some places begins, often followed by ulcerative destruction and waste of the superincumbent soft parts, until the tissues are so soft that they can be pierced by the probe without causing much pain.

Under such circumstances, the pockets filled with pus, rancid juices, decomposed food, microbes, and sharp, irritating, earthy deposits projecting from the root, congestion, stagnation, and disease all about, with little or no nutrient supplies, caries ends in necrosis, often followed by waste of the coverings until finally the dead bone is exposed.

As the condition preceding death of the bone is more apt to interfere with the nutrient supplies of the septums and edges of the sockets than elsewhere, necrosis is shown there first; from the edges of the socket death extends downward, but generally, more rapidly in the septums.

Pus from necrosed tissue is sometimes, not always, of an ichorous nature, which, mingled with the juices in the pockets, if allowed to accumulate aggravates to such an extent that the tumefaction takes on the appearance of abscess, a result which sometimes is sufficiently painful to prevent sleep. There is not only tumefaction of the soft tissues, but the bony portions of the sockets often waste away, so that after the deposits are removed there is left a space of one-eighth of an inch.

Fortunately, necrosis does not often occur in loculosis alveolaris; but when it does, the treatment in addition to that which is necessary in other stages is excision of the dead tissues, and the occasional use of sulphate of zince, thirty grains to the ounce of water.

## Figures and Tables

**Fig. 7. f1:**
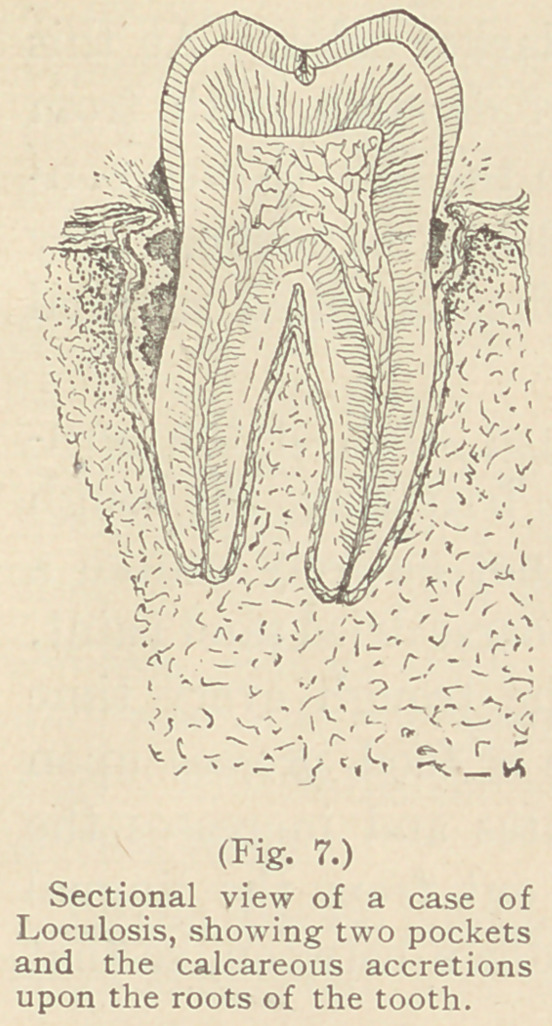


**Fig. 8. f2:**